# Determinants of maternal near miss events among women admitted to tertiary hospitals in Mogadishu, Somalia: a facility-based case–control study

**DOI:** 10.1186/s12884-022-04987-3

**Published:** 2022-08-22

**Authors:** Hassan Abdullahi Dahie

**Affiliations:** 1 Department of Nursing and Midwifery, Faculty of Medicine and Health Sciences, SIMAD University, Mogadishu, Somalia; 2 SOS Children’s Villages, Mogadishu, Somalia

**Keywords:** Maternal near-miss, Pregnancy, Childbirth, Maternity services

## Abstract

**Background:**

A maternal near-miss is a situation in which a woman was on the verge of death but survived a life-threatening obstetric complication that happened during pregnancy, childbirth, or within 42 days after the pregnancy's termination. Survivors of near-miss events share several features with mothers who have died and identifying determinants of maternal near miss will aid in improving the capacity of the health system to reduce severe maternal morbidity and mortality. Therefore, this study was designed to identify determinants of maternal near miss incidents among women hospitalized to tertiary hospitals in Mogadishu, Somalia.

**Methods:**

A facility-based unmatched case–control study was conducted in four tertiary hospitals in Mogadishu from May 1 to July 31, 2021. A total of five hundred thirty-three (178 cases and 355 controls) study participants were involved in the study. The discharge period, cases were recruited consecutively as they emerged, whereas controls were chosen using systematic sampling approach based on every fifth interval of those delivered through normal spontaneous vaginal delivery. Women who were hospitalized during pregnancy, delivery, or within 42 days of termination of pregnancy and met at least one of the maternal near-miss disease specific criteria were classified as cases, while women who were admitted and gave birth by normal vaginal delivery and resealed from the hospital without experiencing severe obstetric complications were considered controls. Participants were interviewed by well-trained research assistants using pre-tested structured questionnaire and the medical records were reviewed to identify maternal near-miss cases. Data were entered into and analyzed with SPSS 25.0. Logistic regression was used, and the significance level was set at *p* value ≤ 0.05.

**Results:**

The most common maternal near-miss morbidities identified were severe anemia (32%), severe pre-eclampsia (19.6%), severe ante partum haemorrhage (15.0%), abortion complications (8.4%), eclampsia (6.1%), ICU admission (5.6%), severe PPH (2.8%) and severe systemic infections (2.8%). The main factors associated with maternal near-miss were rural residency [OR = 2.685, 95%CI: (1.702–4.235)], age below 20 years [OR = 2.728, 95%CI: (1.604–4.5640)], unmarried [OR = 2.18, 2.18, 95%CI (1.247–3.81)], lack of formal education [OR = 2.829, 95%CI: (1.262–6.341)], husband’s unemployment [OR = 2.992, 95%CI: (1.886–4.745)], low family income [OR = 3.333, 95%CI (1.055–10.530)], first pregnancy before 18 years of age [OR = 3.091, 95% CI: (2.044–4.674)], short birth interval [OR = 5.922, 95%CI: (3.891–9.014)], previous history of obstetric complication [OR = 6.568, 95%CI: (4.286–10.066)], never attended ANC services [OR = 2.687, 95%CI: (1.802–4.006)], lack of autonomy in seeking medical help [OR = 3.538, 95%CI: (1.468–8.524)], delivery at non-health facility setting [OR = 4.672, 95%CI: (3.105–7.029)], experiencing the second delay [OR = 1.773, 95% CI: (1.212–2.595)] and stillbirth of the last pregnancy [OR = 5.543, 95%CI: (2.880–10.668)].

**Conclusion:**

and recommendation.

Lack of maternal education, lack of antenatal care, lack of autonomy to seek medical assistance, short birth interval, rural residence and delay in accessing obstetric services were identified as factors associated with maternal near-miss morbidity. As a result, the study suggests that those modifiable characteristics must be improved in order to avoid severe maternal complications and consequent maternal death.

## Background

Pregnancy and childbirth are significant events in woman’s life and a transition to motherhood. These can be times of great hope and blissful expectation. It can also be a period of terror, pain, and even death. Although pregnancy is not an illness but rather a natural physiological process, it is associated with certain health and survival concerns for both the mother and her fetus. These risks are present to a varying degree in every society and in every setting. But they were largely overcome in developed countries, as all pregnant women receive professional care during pregnancy and childbirth. However, such is not the case in many developing countries, where pregnancy is a long journey fraught with dangers that frequently result in health issues or death [1]. This is against the global safe motherhood initiative initiated in 1987 which states that “no woman or foetus or baby should die or be harmed by pregnancy or birth”. Unfortunately, the many women in the developing world die due to pregnancy and childbirth complications [2].

In spite of the fact that maternal mortality remains a serious public health concern, large-scale maternal deaths are uncommon in communities, making evaluations of maternal care quality and consequences difficult [3]. The most devastating end to a pregnant woman is maternal death, and it is sometimes characterized as only the "tip of the iceberg," while maternal morbidity is the "base," and many more will live for every woman who dies, but often suffer from lifelong disabilities [4].

To address this challenge, maternal near miss initiative was introduced into maternal health care to complement the information obtained from maternal deaths reviews [5]. A maternal near-miss (MNM) occurs when a woman is on the verge of dying but survives from a life-threatening obstetric complication that occurred during her pregnancy, childbirth, or within 42 days of termination of pregnancy [6]. This initiative has gained global recognition as an appropriate indicator of maternal care and services [7]. The concept arose from the awareness that women who survive life-threatening health issue associated with pregnancy and childbirth have many characteristics with those who die as a result of such complications. As argued, for every woman who dies, an estimated 20 or 30 more suffer morbidity related to pregnancy and childbirth [3, 8]. Investigating the similitudes, the differences and the relationships between women who died and those who survived life-threatening maternal conditions provide a more comprehensive assessment of maternal health care quality. Moreover, near-misses are more prevalent and statistically robust than maternal death and it helps to identify what goes wrong in pregnancy related care when an MNM occurs in a given setting [9, 10].

Over two decades of instability and the chronic crisis situation in Somalia have had a negative impact on access to healthcare and professional development, including reproductive health services [11]. Being among the low resource setting countries with poor health infrastructure, Somalia has been unable to lower the maternal death rate, which has prevented it from meeting the MDGs [12]. Despite the fact that girls in Somalia have a one in 22 lifetime risk of maternal demise making the maternal mortality rates in the country among the highest in the world [13], factors associated with these maternal risks have not been adequately studied. As a result, the aim of this study was to determine the factors that influence maternal near-miss events in Somalia.

## Methods and materials

### Study setting, period and design

This was a facility-based case–control study conducted in four purposively selected tertiary hospitals in Benadir region-Somalia from May 1 to July 31, 2021 using WHO criteria for maternal near miss. Benadir is a Somali administrative region (gobol) in the southeast. It encompasses the same territory as Mogadishu which is the country's capital and most populous city. The region has 10 tertiary hospitals with well-organized laboratory and surgical capacities for comprehensive emergency obstetric and newborn care.

Four hospitals namely SOS Mother & Child Hospital, Benadir Hospital, De Martini Hospital and Mogadishu Somali Türkiye Training and Research Hospital, were purposively selected from the 10 hospitals in the region based on the number of healthcare workforce, number of deliveries conducted annually and the availability of comprehensive emergency obstetric and newborn care services (CEmONC). These hospitals have over 200 qualified healthcare cadres in their maternity departments and perform over 12,000 deliveries every year. They operate as referral hospitals, are the main providers of comprehensive obstetric and newborn care services in Benadir region and across the country.

### Study populations

Women who were admitted to the selected hospitals during pregnancy, labor, or within the first 42 days of termination of pregnancy from May 1 to July 31, 2021 were considered as the study population.

### Selection of the cases

Cases were women who were admitted to the hospitals for pregnancy-related complications, labour/ abortion, or within 42 days of termination of pregnancy. As in line with the WHO's maternal near-miss inclusion criteria, any women experiencing at least one of the severe maternal complications (obstructed labor, obstetric hemorrhage, pregnancy-induced hypertension disorders, sepsis and severe anemia), been admitted to intensive care unit for critical interventions (interventional radiology, laparotomy or use of blood products) or developed a life-threatening condition leading to system dysfunction (cardiovascular, renal, hepatic, respiratory, haematological and neurological dysfunctions) were taken as cases (Table 1). Thus, cases were recruited consecutively as they appeared. Women who developed complications after 42 days of termination of pregnancy and mothers whose medical records missed important variables were excluded from the present study [14, 15].

**Table 1:** Inclusion criteria for near miss mother (WHO)

Severe maternal complications	Critical interventions or intensive care unit use
Severe postpartum hemorrhage Severe pre-eclampsia or eclampsia Sepsis or severe systemic infection Ruptured uterus Severe complications of abortion	Admission to intensive care unit Interventional radiology Laparotomy (includes hysterectomy, excludes caesarean section) Use of blood products
**Life-threatening conditions (near-miss criteria)**	
**Cardiovascular dysfunction** Shock, cardiac arrest (absence of pulse/heart beat and loss of consciousness), use of continuous vasoactive drugs, cardiopulmonary resuscitation, severe hypoperfusion (lactate > 5 mmol/l or > 45 mg/dl), severe acidosis (pH < 7.1)	**Respiratory dysfunction** Acute cyanosis, gasping, severe tachypnea (respiratory rate > 40 breaths per minute), severe bradypnea (respiratory rate < 6 breaths per minute), intubation and ventilation not related to anesthesia, severe hypoxemia (O_2_ saturation < 90% for ≥ 60 min or PAO_2_/FiO2 < 200)
**Renal dysfunction** Oliguria non-responsive to fluids or diuretics, dialysis for acute renal failure, severe acute azotemia (creatinine ≥ 300 µmol/ml or ≥ 3.5 mg/dl)	**Coagulation/haematological dysfunction** Failure to form clots, massive transfusion of blood or red cells (≥ 5 units), severe acute thrombocytopenia (< 50 000 platelets/ml)
**Hepatic dysfunction** Jaundice in the presence of pre-eclampsia, severe acute hyperbilirubinemia (bilirubin > 100 µmol/l or > 6.0 mg/dl)	**Neurological dysfunction** Prolonged unconsciousness (lasting ≥ 12 h)/coma (including metabolic coma), stroke, uncontrollable fits/status epilepticus, total paralysis
**Uterine dysfunction** Uterine hemorrhage or infection leading to hysterectomy	

Adopted from WHO (2011)

### Inclusion criteria of cases

#### Selection of controls

All women admitted to selected tertiary hospitals during pregnancy, labour, or within the first 42 days after giving birth, and who did not have any of the complications specified in the WHO near-miss criteria, were considered as a source population for control [15]. The study population for controls consisted of those selected mothers from the maternity wards of the selected hospital and discharged without any of the aforementioned medical complications. On the other hand, those mothers who were initially assigned to the control group and discharged normally but unfortunately readmitted with a complication, and women whose medical records missed important variables were excluded from the study.

### Sample size determination

Sample size determination for unpaired case–control studies was used to determine the sample size employing Epi Info 7.0 software. During the estimation of the sample size, the following assumptions were considered: 95% confidence level, 80% power, the ratio of the case to control 1:2, the percent of control exposed and case exposed were 33.3%, and 14.1%, respectively and AOR of 3.25 from previous study in neighboring Ethiopia which revealed that lack of antenatal care (ANC) was one of the significant determinant variables for maternal near miss [16]. Based on these assumptions, the estimated sample size was 483 (161 cases and 322 controls). After considering the non-response of 10%, the final sample size used for this study was 533(178 cases and 355 controls). The case to control group ratio was 1:2, meaning that for each MNM case, two participants from the control groups were recruited.

### Sampling procedures

Four hospitals were chosen through purposive sampling out of ten tertiary hospitals in the Benadir region based on the number of healthcare workers, the number of deliveries performed annually, and the availability of complete emergency obstetric and newborn care services (CEmONC). The sample size for each hospital was proportionally distributed based on their client flow as follows: Benadir Hospital (51 cases and 101 controls), Türkiye Training and Research Hospital (48 cases and 96 controls), SOS Mother & Child Hospital (43 cases and 86 controls) and De Martini Hospital (36 cases and 72 controls).

Cases were then recruited consecutively as they appeared, whereas for every near-miss case selected, two (2) controls were chosen using systematic random sampling approach based on every fifth interval of those delivered through normal spontaneous vaginal delivery on discharge. The 5^th^ interval was found by dividing the average number of controls who visited each hospital in the previous three months by the proportionally allotted control sample size for each hospital [17].

### Data collection tool, procedure, and personnel

The data for this study was obtained between May 1 to July 31, 2021 using a structured questionnaire, as well as near-miss data abstraction methods from a variety of sources, were employed [5, 18]. The information was collected from maternity departments of the selected hospitals using WHO’s abstraction checklist to identify maternal near-miss incidents [6]. The diagnoses of maternal near miss cases and controls were made by general practitioners, obs-gyn physicians, and certified midwives working for the designated institutions, and data was collected from medical records of the cases and controls by well-trained data collectors of nursing and midwifery background. The selected cases and controls were interviewed by the data collectors using the structured questionnaire to get the required information. The data collection tool consisted of 32 items categorized into three main sections namely: -sociodemographic characteristics, obstetric characteristics and healthcare services related characteristics.:

#### Demographic characteristics

Place of residence, age, marital status, level of education, occupation, husband’s education level, income and family size were considered as demographic variables.

#### Obstetric characteristics

Age at first pregnancy, gravidity (the number of times that a woman has been pregnant), parity (the number of times a woman has given birth to a live neonate) and history of obstetric complications (experience of the woman with a major medical condition related to pregnancy, childbirth & puerperium) [19].

#### Healthcare services related characteristics

Antennal care attendance (ANC) attendance, number of ANC attendance, timing of ANC attendance, place of last ANC visit, place of last birth, mode of delivery, history of cesarean section, birth preparedness, circumstances of birth preparation, knowledge of pregnancy danger signs, referral type, means of transportation, decision to seek health services and the 3 maternal delays: The first delay occurs when the time between identifying health problems and deciding to seek maternal health care exceeds 24 h, the second delay occurs when the time between deciding to seek health care and arriving at the facility exceeds one hour, and the third delay occurs when the time between arriving at the facility and accessing services exceeds one hour [20].

### Data quality assurance procedure

Before the actual data collection, senior qualified nursing-midwives who fluently speak in English and Somali languages were selected as data collectors and then trained for two days about the data study objectives, interviewing techniques, identification and selection of cases & controls, data collection tools, ethical issues, responsibilities of data collectors and the techniques of research quality control. The pretest was done on 5% of the sample size (9 cases and 18 controls) on similar maternity hospital in the region that was not sampled for the study, with necessary modifications from the feedback incorporated accordingly. Following the pre-testing the questionnaire, Cronbach’s Alpha was calculated using SPSS window version 25.0 and obtained a Cronbach’s Alpha of 0.89, indicating that items were fit for purpose [21]. During data collection, regular supervision was done by the principal investigator.

Similarly, the soft copies of data collected were coded and the hard copies locked in a locker in the office of the principal researcher. The data collection team had access to data only when permission was granted by the principal researcher. The returned and completed tools have been cross-checked by the principal researchers to ascertain their completeness. Questionnaires with missing data were re-administered to the respondents for correction if the respondent was still available or the missing data could be found in patient admission card.

### Ethical approval and consent to participate

Ethical clearance was obtained from the institutional review board (IRB) of SIMAD University, faculty of medicine and health sciences. Written permission was also obtained from the study hospitals. Similarly, all the individuals identified for recruitment into the study were informed of the study's purpose. Subsequently, verbal informed consent was obtained from all the individuals willing to participate in the study before answering the questionnaires through the research assistants. They were also informed that their involvement was entirely voluntary, and they were free to withdraw at any moment if they so desired. They were guaranteed that there was no victimization for refusal & full privacy and confidentiality.

### Data analysis and management

The data were entered into, cleaned and analyzed using SPSS version 25.0. Univariate and bivariate analysis: proportions, frequencies, averages and crosstabulation were calculated for study variables to compare cases and controls. Binary logistic regression was used to identify predictor variables for maternal near miss. A *p* < 0.05 was considered significant.

## Results

### Socio-demographic characteristic

A total of 533 participants (178 cases and 355 controls) were interviewed, with a 100% response rate. The mean age (± SD) for cases and controls was 24.56 (± 7.49) and 29.04 (± 5.79) years, respectively. About 12.4% of the controls and 27.5% of the controls were rural dwellers. Regarding maternal education, 72% of the cases and 50% of the controls had not formal education. A significant proportion of the cases (15.2%) were daily laborers and breadwinners of their respective families whereas as less than 4% of the controls were daily laborers.

According to the findings, 5.9% of the controls and 11% of cases were divorced. No big difference was found with respect to family income, with 94.4% of the cases and 91.5% of the controls were living with less than 500USD per month.

Concerning the determinant factors, the study also showed that maternal near was significantly associated with the residence, age, marital status, maternal education, husband’s employment and the family size. The odd of maternal near-miss occurrence was 2.7 times higher among rural dwellers than urban residents [OR = 2.685, 95%CI: (1.702–4.235), *p* < 0.001]. Similarly, regarding maternal age, the odd of maternal near miss was 2.7 times higher among those younger than 20 years compared to those whose age is between 20–35 years [OR = 2.728, 95%CI: (1.604–4.5640), *p* < *0.001*]. According to study findings, respondents who were not in marriage contract (i.e. divorced or widowed) at the time of the study were two times more likely to have maternal near miss event compared to those in marriage contract [OR = 2.18, 95%CI: (1.247–3.81), *p* < *0.05*]. Moreover, the odds of maternal near miss were 2.8, 3.0, 3.3 and 1.5 times higher among women without formal education were (OR = 2.829, 95%CI: (1.262–6.341), *p* < *0.05*], women whose husband were unemployed [OR = 2.992, 95%CI: (1.886–4.745), *p* < *0.001*], women with monthly family income < 100 USD [OR = 3.333, 95%CI: (1.055–10.530), *p* < *0.05*] and women with family size comprising of ≤ 5 members [OR = 1.530, 95%CI: (1.042–2.247), *p* < *0.05*], respectively. Other demographic variables considered were not statistically significant as shown in Table 2.

**Table 2:** Socio-demographic characteristic of respondents

		Maternal Near-miss status			
Socio-demographic Characteristics	N (%)	Near-miss (*n* = 178)	Non-near-miss (*n* = 355)	Odds Ratio (95% Cl)	*P* Value
Residence					
Urban	440 (82.5)	129(72.5)	311(87.6)	Ref	
Rural	93(17.5)	49(27.5)	44(12.4)	2.685(1.702–4.235)	0.0001*
Mother’s Age (years)					
< 20 years	65(12.2)	35(19.7)	30(8.5)	2.728(1.604–4.5640)	0.0001*
> 35 years	54(10.1)	19(10.7)	35(9.9)	1.270(0.699–2.306)	0.433
20–35 years	414(77.7)	124(69.7)	290(81.7)	Ref	
Marital Status					
Married	477(89.5)	150(84.3)	327(92.1)	Ref	
Unmarried	56(10.5)	28(15.7)	28(7.9)	2.18 (1.247–3.81)	0.006*
Mother’s education					
No formal education	309(58.0)	128(71.9)	181(51.0)	2.829 (1.262–6.341)	0.012*
Primary education (1-8^th^)	121(22.7)	30(16.9)	91(25.6)	1.319 (0.548–3.172)	0.537
Secondary(9-12th)	63(11.8)	12(6.7)	51(51.4)	0.941 (0.347–2.553)	0.905
College and above	40(7.5)	8(4.5)	32(9.0)	Ref	
Husband’s Education					
No formal education	201(37.7)	89(50.0)	112(31.5)	1.987 (1.234–3.199)	0.005*
Primary education (1-8^th^)	84(15.8)	31(17.4)	53(14.9)	1.462 (0.812–2.633)	0.205
Secondary(9-12th)	122(22.9)	22(12.4)	100(28.2)	0.550 (0.301–1.004)	0.052
College and above	126(23.6)	36(20.2)	90(25.4)	Ref	
Maternal employment status					
Employed	92(17.3)	36(20.2)	56(15.8)	1.354(0.851–2.153)	0.201
Unemployed	441(82.7)	142(79.8)	299(84.2)	Ref	
Husband’s employment status					
Employed	442(82.9)	128(71.9)	314(88.5)	Ref	
Unemployed	91(17.1)	50(28.1)	41(11.5)	2.992(1.886–4.745)	0.0001*
Monthly family income					
< 100 USD	19(3.6)	10(5.6)	9(2.5)	3.333(1.055–10.530)	0.040*
100–500 USD	474(88.9)	158(88.8)	316(89.0)	1.500(0.715–3.146)	0.283
> 500 USD	40(7.5)	10(5.6)	30(8.5)	Ref	
Household size					
≤ 5	162(30.4)	65(36.5)	97(27.3)	1.530(1.042–2.247)	0.030*
> 5	371(69.6)	113(63.5)	258(72.7)	Ref	

### Obstetric characteristics of respondents

According to the findings, approximately one-third (30%) of the cases and (9.0%) of the controls married before their 16th birthday, while 37.8% of the cases and 16.3% of the controls had their first pregnancy before turning to 18. The study has also discovered that 31.5% of the cases and 23.9% of the controls had parity of higher than 5, with 66.3% of the cases and 37.2% of the controls having a birth interval of less than 24 months. More interestingly, it has been found that about half (49.4%) of the cases and 12.9% of the controls had a history of pregnancy-related complications.

In terms of determining factors, the study has revealed that maternal near miss events were associated with the age at first pregnancy, mother’s birth interval, previous history of obstetric complication and last birth outcome. The study has also shown that odds of maternal near-miss were 3.1, 5.9 and 6.6 times higher among women who had their first pregnancy before 18 years of age [OR = 3.091, 95% CI: (2.044–4.674) *p* < *0.001*], those having birth interval of less than 2 years [OR = 5.922, 95%CI: (3.891–9.014), *p* < *0.001*] and those with previous history of obstetric complications [OR = 6.568, 95%CI: (4.286–10.066) *p* < *0.001*], respectively. On the other hand, however, the study has found that maternal near miss events were not associated with maternal gravidity and parity (Table 3).

**Table 3:** Obstetric characteristics associated with maternal near-miss

			Maternal Near-miss status		
Obstetric Characteristics	N (%)	Near-miss (*n* = 178)	Non near-miss (*n* = 355)	Odds Ratio (95% Cl)	P Value
Age at first Pregnancy (years)					
< 18 years	125(23.5)	67(37.6)	58(16.3)	3.091(2.044–4.674)	0.0001*
≥ 18 years	408(76.5)	111(62.4)	297(83.7)	Ref	
Gravidity					
≤ 3	216(40.5)	73(41.0)	143(40.3)	Ref	
> 3	317(59.5)	105(59.0)	212(59.7)	0.970(0.673–1.399)	0.871
Parity					
0–2	183(34.3)	56(31.5)	127(35.8)	Ref	
3–5	209(39.2)	66(37.1)	143(40.3)	1.047(0.682–1.607)	0.835
> 5	141(26.5)	56(31.5)	85(23.9)	1.494(0.924–2.369)	0.088
Birth interval					
< 24 months	280(52.5)	141(79.2)	139(39.2)	Ref	
≥ 24 months	253(47.5)	37(20.8)	216(60.8)	5.922(3.891–9.014)	0.0001*
Previous history of obstetric complication					
Yes	134(25.1)	88(49.4)	46(13.0)	6.568(4.286–10.066)	0.0001*
No	399(74.9)	90(50.6)	309(87.0)	Ref	

### Healthcare services related characteristics

According to the study, about 80.6% of the controls and 60.7% of the cases received at least one ANC visit, while, almost half (47.2%) of the cases and 16.1% of the controls gave birth at homes. Regarding preparation for birth, it has been found that only 45% of the cases and 80% of the controls had prepared themselves for birth in one way or the other. Similarly, the study had shown that less than half for both cases and controls had basic knowledge for danger signs of pregnancy. Moreover, only 3.4% of the cases and 11.0% of controls had freedom for seeking medical help and more than half (55.5%) of the controls and 69.1% of cases had unfortunately taken more than one hour to reach the nearest health facility. On the other, 81.5% of the cases and 96.1% of the controls had live birth outcome.

With respect to the association between respondent’s healthcare service characteristics and maternal near miss events, the study has identified that odd of maternal near miss events were associated with ANC attendance, means of transportation, referral point, autonomy in seeking medical help, delays and birth outcomes.

In terms of ANC attendance, odds of maternal near-miss occurrence were 2.7 times higher among women who never received ANC services [OR = 2.687, 95%CI: (1.802–4.006), *p* < *0.001]*.

Similarly, the odds of maternal near events were 4.5, 4.7, 4.5, 3.5, 1.8 and 5.5 times higher among women who never prepared for birth [OR = 4.5, 95%CI:(3.058–6.688), *p* < *0.001*], mothers giving birth in non-health facility setting compared to those who gave birth in health facilities [OR = 4.672, 95%CI: (3.105–7.029), *p* < *0.001*], self-refers [OR = 4.479, 95%CI: (2.777–7.223), *p* < *0.001*], women who were non-autonomous in seeking medical help [OR = 3.538, 95%CI: (1.468–8.524), *p* < *0.005*], women who took more than an hour to reach health facility [OR = 1.773, 95% CI: (1.212–2.595), *p* < *0.005*]. Furthermore, the incidences of maternal near misses were 5.543 times higher among those women whose last birth outcomes was stillbirth [OR = 5.543, 95%CI: (2.880–10.668), *p* < *0.001*], (Table 4).

**Table 4:** Healthcare services related characteristics

		Maternal Near-miss status			
Maternal health service-related characteristics	N (%)	Near-miss (n = 178)	Non-near-miss (n = 355)	Odds Ratio (95% Cl)	P Value
ANC^*^ Attendance					
Yes	394(73.9)	108(60.7)	286(80.6)	Ref	
No	139(26.1)	70(39.3)	69(19.4)	2.687(1.802–4.006)	< 0.001*
Birth Preparedness					
Yes	364(68.3)	82(46.1)	282(79.4)	Ref	
No	169(31.7)	96(53.9)	73(20.6)	4.523(3.058–6.688)	< 0.001*
Place of Last Delivery					
Health facility	392(73.5)	92(52.8)	298(83.9)	Ref	
Home	141(26.5)	84(47.2)	57(16.1)	4.672(3.105–7.029)	< 0.001*
Knowledge of danger signs					
Yes	258(48.4)	93(52.2)	165(46.5)	1.260(0.879–1.807)	0.209
No	275(51.6)	85(47.8)	190(53.5)	Ref	
Means of transportation					
Ambulance	45(8.4)	25(14.0)	20(5.6)	1.176(0.478–2.897)	0.724
Private transport	326(61.2)	89(50.0)	237(66.8)	0.353(0.171–0.730)	0.005*
On foot	129(24.2)	47(26.4)	82(23.1)	0.539(0.249–1.166)	0.117
Other	33(6.2)	17(9.6)	16(4.5)	Ref	
Referred by					
Self	444(83.3)	122(68.5)	322(90.7)	4.479(2.777–7.223)	< 0.001*
Health facility	89(16.7)	56(31.5)	33(9.3)	Ref	
Autonomy in seeking medical help					
Yes	45(8.4)	6(3.4)	39(11.0)	Ref	
No	488(91.6)	172(96.6)	316(89.0)	3.538(1.468–8.524)	0.005*
Fist Delay (decision to seek medical help took > 24 h)					
Yes	31(5.8)	13(7.3)	18(5.1)	1.475(0.706–3.083)	0.301
No	502(94.2)	165(92.7)	337(94.9)	Ref	
Second Delay (time to reach to the facility took more than 60 min)					
Yes	321(60.2)	123(69.1)	198(55.8)	1.773(1.212–2.595)	0.003*
No	212(39.8)	55(30.9)	157(44.2)	Ref	
Third Delay (time to receive required health service took 60 min)					
Yes	423(79.4)	138(77.5)	285(80.3)	1.180(0.761–1.830)	0.459
No	110(20.6)	40(22.5)	70(19.7)	Ref	
Last birth outcome					
Live birth	486(91.2)	145(81.5)	341(96.1)	Ref	
Stillbirth	47(8.8)	33(18.5)	14(3.9)	5.543(2.880–10.668)	< 0.001*

^*^ANC Ante natal care

### Distribution of severe maternal morbidity among near miss events

**Fig. 1:**
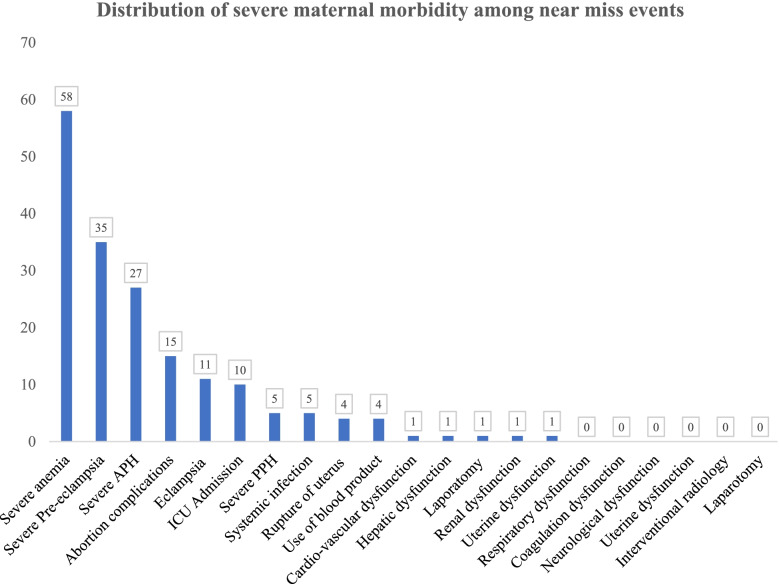
Distribution of maternal near miss events

The study has identified that the most common cause of maternal near misses was severe anemia (32.4%) followed by severe pre-eclampsia (19.6%), severe ante-partum haemorrhage (15.0%), abortion complications (8.4%) and eclampsia (6.1%). Similarly, the study has revealed that 5.6%, 2.8%, 2.8%, 2.2%, 2.2%, 2.2%, 0.6%, 0.6%, 0.6% and 0.6% of the maternal near miss cases were due to ICU admission, severe post-partum haemorrhage, systemic infection, rupture of uterus, use of blood products, cardio-vascular dysfunction, hepatic dysfunction, laparotomy, renal dysfunction and uterine dysfunction, respectively (Fig. 1).

## Discussion

Women in Somalia face one of the greatest lifetime risks of maternal death in the world, with a one in 22 lifetime risk of maternal death. These lifetime risks are the very main causes of maternal mortality in Somalia, which is among the highest in the world [22]. In order to tackle maternal deaths, clear understanding of the magnitude and associated factors of maternal near miss in a given setting is crucial [23]. The study has identified that more than 80% of maternal near misses were severe anemia, severe pre-eclampsia, eclampsia, severe ante partum haemorrhage and abortion complications. This was almost in line with several studies conducted in Harare [24, 25].

Most surprisingly, this study revealed that severe anemia was the leading cause of maternal near-misses. Although this result is quite contrary to most of the studies, it is consistent with a study conducted in Ethiopia which found almost similar results [26]. This may be explained by a number of factors, the first of which is that due to the country's poor health system, most pregnant women do not receive antenatal care. Secondly, many pregnant & lactating women do not have adequate nutrition due low economic profile. Thirdly, there is a common belief in the Somali community that if a pregnant mother eats a well-balanced diet, her baby will grow up and therefore be unable to give birth normally, necessitating surgery. This lead that the mothers deliberately avoid nutritious foods, resulting in severe anemia.

This study identified numerous sociodemographic, obstetric and healthcare factors associated with maternal near misses. Regarding socio-demographic characteristics, it has been found that residence, age, marital status, education, employment and family size were significantly associated with maternal near miss events. It has been revealed that mothers residing in the rural were almost three times more likely to have a maternal near miss event compared to mothers who resided in the urban [OR = 2.685, 95%CI: (1.604–4.5640), *p* =  < 0**.**0001]. This can be explained by the fact that majority of the pregnant women in rural areas do not have access to basic maternity services.

Regarding the maternal age, the study found that odd of maternal near miss was almost three times higher among those younger than 20 years compared to those between 20–35 years of age [OR = 2.728, 95%CI:(1.604–4.564). The findings of this study are consistent with other studies conducted in Brazil, Ethiopia and Lao which all found that young age was an important predictor of maternal near miss [27–29]. This can be explained that young women are more susceptible to childbearing complications such as anemia in pregnancy, pregnancy-induced hypertensions, preterm labour, abortions, septicemia and so on. In addition, many young Somalis marry secretly without the knowledge of the families & communities or the pregnancy may occur outside the context of proper marriage, exposing the young women to adverse social consequences. Furthermore, many young Somalis marry surreptitiously without their families' or communities' knowledge, or pregnancies may occur outside of the framework of a traditional marriage, exposing young women to negative societal implications. Early marriage and pregnancy are well-known social issues that have been widely discussed in a variety of social and health forums, but they continue to exist in the community, particularly in rural regions and among low-income families.

According to study findings, women who were not in marriage contract at the time of the study were two times more likely to have maternal near miss event compared to those in marriage contract [OR = 2.18, 95%CI, (1.247–3.81, *p* = 0.006)]. The finding of this study regarding marriage contract is consistent with another study conducted in Addis Ababa, Ethiopia which found almost similar result [26].

Moreover, the study has revealed that the odds of developing maternal near-miss events among women with family income of less than 100 USD were 3.33 times more compared to those with more than 500 USD [OR = 3.333, 95%CI = (1.055–10.530)]. The result of this study was consistent with other study conducted in Ethiopia a year ago divulged that women with the lowest monthly income (1000 ETB) had 3.99 times the chance of having maternal near-miss than those with a monthly income more than or equal to 3001 ETB [30]. This is not surprising since over 99% of maternal deaths occur in low- and middle-income countries due to extreme poverty resulting in lack of access to quality healthcare and education of women [2]. The magnitude of maternal near miss varies between and within countries; however, the highest rates are found in low- and middle-income countries [31–, 32–34]. Education increases women’s access to relevant information and may facilitate the financial means required to pay for transportation to care [35].

Regarding obstetric factors, the study revealed that maternal near miss events were associated with the maternal age at first pregnancy, mother’s birth interval, previous history of obstetric complication and last birth outcome.

This has found that age at first pregnancy were significant predictor of maternal near-miss occurrence. The odd of maternal near-miss occurrence was 3.4 times higher among women who had married at the age of 15 years or earlier than those aged twenty-one years (OR = 3.4, 95%CI, 1.912–6.091). On the other hand, when one's age approached 35, there was a growing odd [27, 36]. The observed association between first pregnancy and maternal near miss is consistent with another study in Ethiopia [35].

The study has also shown that odds of maternal near-miss were 3.1 times higher among women who had their first pregnancy before 18 years of age compared to those who had their first pregnancy after 18 years of age (OR = 3.091, 95% CI, 2.044–4.674). Almost similar study results were presented by several studies [17, 37, 38]. This could be explained that younger women are often not physically capable of childbearing. Furthermore, girls married early are more likely to experience violence, abuse and forced sexual relations due to unequal power relations, exposing women to adverse social consequences. They are also vulnerable to sexual transmitted infections and severe pregnancy symptoms. The study has also shown that the odds of a maternal near-miss were six times higher among women with a birth interval of less than two years compared to those with a birth interval of more than two years [OR = 5.922, 95%CI, (3.891–9.014)]. This is in line with several studies conducted in several counties in East Africa [17, 39, 40].

Another important predictor was those with previous history of obstetric complications. This showed that the women with previous history obstetric complications were 6.5 times more likely to have maternal near miss than those without history of obstetric complications [OR = 6.568, 95%CI, (4.286–10.066]. Several studies found the almost same results [35, 41]. On the other hand, contrary to study conducted in Bangladesh, the study has found that maternal near miss events were not significantly associated with maternal gravidity and parity [41]. This can be attributable to many factors, including differences in the socio-economic situations and healthcare systems of the two countries.

With respect to the association between respondent’s healthcare service characteristics and maternal near miss events, the study has identified that odd of maternal near miss events were associated with ANC attendance,, means of transportation, referral point, autonomy in seeking medical help, delays and birth outcomes.

In terms of ANC attendance, the odds of maternal near-miss occurrence were 2.7 times higher among women who never received ANC services [OR = 2.687, 95%CI, (1.802–4.006)]. This finding is consistent with many other studies conducted in several places in the world ([16, 25, 42]. This finding is consistent with other studies conducted in several countries [17, 35, 43, 44]. This can be explained that utilization of ANC reduces the maternal morbidity and mortality rates by screening high-risk mothers for complications and facilitating a rapid diagnosis and management of life-threatening obstetric conditions [45].

Similarly, the odds of maternal near events were 4.7 times higher among mothers giving birth in non-health facility setting compared to those who gave birth in health facilities [OR = 4.672, 95%CI, (3.105–7.029)]. This is in line with a study conducted in Morocco [44]. The advantages of hospital birth over home delivery are widely established, including adequate pain management, access to a NICU in the event of an emergency, professional staff assistance, and the availability of advanced interventions.

Women who were non-autonomous in seeking medical help were 3.5 times more likely to have maternal near-miss events [OR = 3.538, 95%CI, (1.468–8.524)]. This is consistent with s study in neighboring Ethiopia [17]. This could be explained that respecting the autonomy of the women allows them to make decisions that are in their best interests, as they are usually the best judges of those interests.

The study discovered that women who took more than an hour to reach health facility were almost two times more likely to be maternal near miss than whose who arrived in less than one hour [OR = 1.773, 95% CI, (1.212–2.595)]. Although statistically insignificant, the other two delays also exhibited a greater odd. The findings of this study correspond to the results of other studies conducted in some parts of Africa like Ethiopia and Morocco [17, 44]. Delays in obtaining care were collected according to the 3-delay model of Thaddeus and Maine, [46], which was adapted as follows. Firstly, delay in decision to seek care as a consequence of women’s low socio-economic status, lack of understanding of life-threatening complications and risk factors in pregnancy and when to seek medical help, previous poor experience of health care and financial implications. Secondly, delay in reaching care due to; distance to health centres and hospitals, unavailability of transportation, lack of cost of transportation, insecurity and poor roads and infrastructure. Thirdly, delay in accessing and receiving adequate health care mainly due to poor facilities and lack of medical supplies, inadequately trained and poorly motivated medical staff and inadequate referral systems [20].

A lot of reasons contribute to this, including poor public infrastructure such as roads, low community awareness, and a health system that is too limited in terms of availability, accessibility, affordability and skilled birth attendants especially in remote areas.

In this study, still birth as the outcome of last pregnancy was positively related to maternal near-miss [OR = 5.543, 95%CI: (2.880–10.668)] and is compatible with studies conducted in Nigeria and Ethiopia [17, 47]. This can be explained by the fact that women with a history of stillbirth may experience a variety of psychological and relational issues, which may raise the risk of maternal complications in subsequent pregnancies. The link between maternal chronic hypertension and stillbirth may also be an alternative explanation. Moreover, the argument of Todd et al. was handy here, in which women who have experienced a stillbirth might have a history of chronic hypertension, and thereby increase the odds of maternal near-miss [48].

### Strength and limitation of the study

The strength of the study is that it is the first of its kind in Somalia to document the determinants of maternal near-miss events among women admitted to tertiary hospitals using the WHO case identification criteria of near-miss mothers. Hense these findings should draw policymaker's attention to the consequences of lack of appropriate obstetric practice and inadequate coverage for emergency obstetric and newborn care..

The selection of women was limited to those who reached the study hospitals. On the other hand, even though the study adapted WHO’s maternal near-miss criteria, due to limited resources, few advanced laboratory investigations and management-based criteria were not used, however, this had no impact on diagnosis and outcome since all other clinical-based criteria were applied.

## Conclusion

In this study, the most common causes of maternal near misses were severe anemia (32%), severe pre-eclampsia (19.6%), severe ante partum haemorrhage (15.0%), abortion complications (8.4%) eclampsia (6.1%), severe post-partum haemorrhage (2.8%) and severe systemic infection (2.8%).

Rural residence, lack of formal education, unemployment, previous history of maternal complications, short birth interval and lack of ante natal care attendance were all found to be significantly associated with maternal near miss cases.

These findings of this study ought to propose in rural areas, particularly those areas high number of unschooled women; emphasizing on maternity service coverage is a critical step in preventing major maternal problems by enhancing healthcare extension packages and strengthening free high-quality antenatal care (ANC) services. Moreover, the study recommends to establish a community awareness program to raise the awareness of the public especially women of reproductive age and address the socio-cultural factors perpetuating early marriage and early pregnancy.

Lastly, the study suggests that a longitudinal multicenter study be conducted to generate a more consistent and complete national picture of maternal near misses.

## Data Availability

Data will be made available upon request. Readers who wish to gain access to the data may write to the corresponding author, Dr. Hassan Abdullahi Dahie, at dahie@simad.edu.so.
